# Risk Perception and Depression in Public Health Crises: Evidence from the COVID-19 Crisis in China

**DOI:** 10.3390/ijerph17165728

**Published:** 2020-08-07

**Authors:** Yubin Ding, Junling Xu, Sisi Huang, Peipei Li, Cuizhen Lu, Shenghua Xie

**Affiliations:** 1 Undergraduate College, Central China Normal University, Wuhan 430079, China; ybding@ccnu.edu.cn; 2 College of Public Administration, Central China Normal University, Wuhan 430079, China; jling.xu@ccnu.edu.cn (J.X.); sisi.huang@mails.ccnu.edu.cn (S.H.); peipei.li@mails.ccnu.edu.cn (P.L.); cuizhen.l@mails.ccnu.edu.cn (C.L.)

**Keywords:** risk perception, depression, public health crisis, COVID-19

## Abstract

*Background:* Scant attention has been paid to how risk perceptions of public health crises may affect people’s mental health. *Aims*: The aims of this study are to (1) construct a conceptual framework for risk perception and depression of people in public health crises, (2) examine how the mental health of people in the crisis of Coronavirus Disease 2019 (COVID-19) is affected by risk perception and its associated factors, including distance perception of the crisis and support of prevention and control policies, and (3) propose policy recommendations on how to deal with psychological problems in the current COVID-19 crisis. *Methods*: Online questionnaire survey was implemented. A total of 6373 people visited the questionnaire online, 1115 people completed the questionnaire, and the number of valid questionnaires was 1081. Structural equation modeling was employed for data analysis. *Results*: Risk perception and its associated factors significantly affect the mental health of people in public health crises. Specifically, (1) distance perception of public health crises is negatively associated with depression among people, (2) affective risk perception is positively associated with depression of people in public health crises, (3) cognitive risk perception is negatively associated with depression of people in public health crises, and (4) support of prevention and control policies is negatively associated with depression of people in public health crises. *Conclusion*: The findings of this study suggest that risk perception plays an important role in affecting the mental health of people in a public health crisis. Therefore, health policies aiming to improve the psychological wellbeing of the people in a public health crisis should take risk perception into consideration.

## 1. Introduction

How crisis events affect mental health has been widely studied by academia [1,2,3,4,5,6,7,8]. Because crisis events may change people’s daily lives or even threaten their safety, the mental health of people suffering from such crises should be particularly noted. Currently, the world is experiencing a serious public health crisis: the spread of COVID-19. Because of the significant threat of this virus, for the first time in human history, the Chinese government decided to lock down Wuhan City from 23 January to 8 April 2020. This strategy was followed by many countries as the virus spread globally. To date, this public health crisis has had a significant impact on the global population. For example, since the outbreak of the virus, more than 4 million people have been infected, and nearly 500,000 people had died of infection by 28 June 2020 [9]. Furthermore, the World Bank predicted that the world economy will shrink by 5.2% in 2020 because of this pandemic [10].

The mental health of the public might be affected by the outbreak of COVID-19. Previous studies have investigated the effect on mental health of crisis events such as economic crises, natural disasters, and war and terrorist attacks [3,4,6,7,11,12,13,14,15,16,17]. However, the effect of public health crises on mental health has not received sufficient attention. With the large number of casualties and significant economic losses resulting from the COVID-19 crisis, people who experience it may suffer great psychological distress. Furthermore, lockdown and quarantine policies cause substantial disturbance to people’s daily lives. Therefore, it is important to understand how these policies affect the mental health of people during this public health crisis. Under such circumstances, the aims of this study are to (1) construct a conceptual framework for risk perception and depression of people in a public health crisis, (2) examine how the mental health of people in the COVID-19 crisis is affected by risk perception and its associated factors, including distance perception of the crisis and support of prevention and control policies, and (3) propose policy recommendations on how to deal with psychological problems in the current COVID-19 crisis.

The remainder of this paper is organized as follows: Section 2 reviews previous studies on the ways in which economic crises, natural disasters, and war and terrorist attacks affect mental health. Section 3 presents a theoretical framework of risk perception and the mental health of people in a public health crisis. Section 4 explains the research design. Section 5 presents the results of structural equation modeling on risk perception and mental health based on online survey data. Section 6 summarizes the main findings of this study and provides theoretical reflections and policy implications.

## 2. Literature Review

How crisis events may affect mental health has been widely examined by researchers globally. Previous studies have focused on the effects of three types of crisis events: economic crises, natural disasters, and war and terrorist attacks. Similar to the COVID-19 crisis, these three types of crisis usually cause huge economic losses or human casualties in a relatively short period of time. Thus, crises such as these usually have a negative impact on the psychological wellbeing of people. However, since the three types of crisis events are different in terms of degree of harm, scope of influence, and duration, the ways that different crisis events affect the mental health of people are also distinctive.

An economic crisis is a type of crisis that may typically be associated with mental health. Economic crises cause incomes to fall and unemployment, thus leading to a decline in living conditions. Researchers have shown that socioeconomic factors are related to happiness and the subjective wellbeing of people [18,19,20]. If a person’s standard of living declines sharply in a short period during an economic crisis, their mental health could be worse than prior to the crisis. Studies show that the mental health of people suffers in economic crises [8,12]. However, this decline only lasts for a short period. After a stage of adaptation, people’s mental health during an economic crisis returns to the level that prevailed before the crisis [21,22].

Natural disasters are the second main type of crisis that may affect people’s mental health. Natural disasters such as tsunamis, earthquakes, floods, and volcanic eruptions threaten people’s lives and property safety. Moreover, disasters are usually difficult to predict. They often occur suddenly. Thus, the mental state of people is greatly stimulated in a short period of time. Some people may experience mental breakdown because of overstimulation. Studies of the 2004 tsunami in Southeast Asia, the 2008 Wenchuan earthquake, and the 2011 earthquake of the Pacific coast of Tōhoku all show that the mental health of people impacted by these natural disasters worsened significantly [2,3,11,17,23,24,25,26]. In addition, people’s mental health during these natural disasters may vary by gender, socioeconomic status, and age [2,3,17]. Moreover, social support and policy support may alleviate the mental distress of people suffering from natural disasters [2].

War and territorial attacks are the third type of crisis that is possibly associated with people’s mental health. In the present day, humans live in a world with uncertainties. War and terrorist attacks may put people at risk at any time. The September 11 attacks is a typical example. The incident resulted in a large number of casualties and caused great economic losses. Nearly 3000 people were killed and, in New York city after the attacks, more than 140,000 jobs were lost in the first month, and $2.8 billion in wages in the first three months [27]. A number of studies have shown that people who experienced the September 11 attacks have mental health problems [15,16,28]. Other studies have also reached similar conclusions based on people who also experienced war or territorial attacks [14]. Furthermore, the negative effect of war and territorial attacks on mental health could last for a long time. For instance, Wang and Yang showed that the mental health problems of people who experienced the September 11 attacks also resulted from the effect of an external traumatic event [28].

Although previous studies have paid significant attention to the association between crisis events and mental health, minimal attention has been paid to the relationship between public health crises and mental health. Given the great influence of COVID-19 on people globally, investigating how COVID-19 may affect the mental health of people is an urgent issue. Precious studies have investigated how the COVID-19 crisis, particularly the lockdown and quarantine policies, affects people’s mental health [1,29,30,31,32,33,34,35,36,37,38,39]. However, few studies have shown how the risk perception of COVID-19 may affect people’s mental health.

Accordingly, this study sought to explore the effect of risk perception and its associated factors on depression in the COVID-19 crisis. This study is different from previous studies because it treated risk perception of public health crises as a potential factor of mental health problems. In addition, because risk perception is affected by distance perception of public health crises, this study included distance perception in its theoretical framework. Moreover, since prevention and control policies play a vital role in preventing the spread of COVID-19, we incorporated the support of prevention and control policies in the theoretical framework. Using online survey data, hypotheses developed by the theoretical framework were empirically tested. The research findings of this study may contribute to understanding the effect of the public health crisis on mental health in both theory and practice.

## 3. Conceptual Framework and Hypotheses

Previous research has substantially explored how crisis events may affect mental health, particularly how demographic factors, income, employment, and social support affect mental health. However, in addition to the influence of these factors, the mental health of people involved in the public health crisis may be related to risk perception and the factors associated with it. For example, in the COVID-19 crisis, how the virus spreads may be not very clear, especially at the early stage of the crisis. People generally experience anxiety and restlessness in an uncertain environment. Thus, how people perceive the danger of a public health crisis could be significantly related to their mental health. Moreover, the distance that a public health crisis spreads is a significant factor that affects the relationship between risk perception and mental health [40]. Therefore, people who are closer to the center of the crisis are more likely to be influenced by the crisis. Additionally, because of the significant role of social and policy support in alleviating the detrimental effect of the disaster on mental health [2], measures taken by the government to respond to public health crises affect people’s sense of security. In a public health crisis, effective government policies that improve people’s sense of security are more likely to be supported by the public [41]. In addition, an increase in the public’s sense of security can reduce depression. Therefore, support for prevention and control policies could be significantly associated with the mental health of the public.

The above analysis suggests that there are three core elements in a public health crisis that may affect people’s mental health: distance perception, risk perception, and policy support. Risk perception is defined as people’s subjective judgments of the possible negative consequences of an event [42]. According to relevant research, risk perception can be divided into two categories: affective risk perception and cognitive risk perception [43,44,45]. Affective risk perception is a type of heuristic information processing that is fast, intuitive, parallel, and spontaneous, and requires little use of cognitive resources. In contrast, cognitive risk perception represents analytical information processing that is slow, cautious, sequential, and controllable, and requires the use of more cognitive resources [46]. Because of the large differences between affective risk perception and cognitive risk perception, we incorporated both in the analytical framework of this study. Distance perception can be defined as how far people think that they are from the center of the crisis. Since distance perception affects the risk perception of a crisis [40], it is a significant factor that should be considered in analyzing the relationship between risk perception and people’s mental health. Policy support refers to the extent to which people support policies implemented by the government. Previous studies have shown that risk perception of a public health crisis is associated with policy support [41]. Usually, when people believe that government responses can effectively cope with the public health crisis, they are more likely to support government policies. Moreover, theoretically, effective policies can strengthen people’s sense of security, thereby helping to reduce anxiety and restlessness. Thus, policy support should be considered in analyzing the relationship between risk perception and the mental health of people.

**Figure 1:**
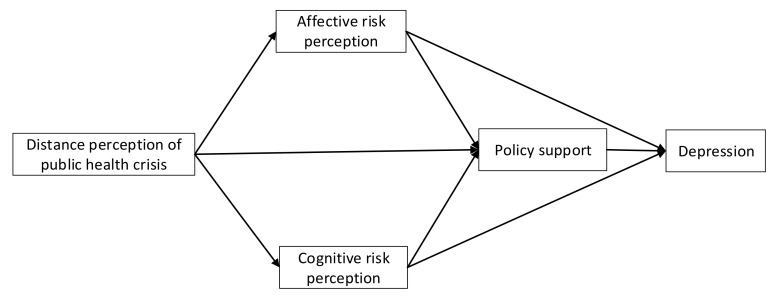
Conceptual framework of depression in a public health crisis.

Figure 1 shows the conceptual framework of depression in a public health crisis. Because the links of the five variables are shaped like a diamond, we named this model the diamond model. According to this model, distance perception is the starting point of how a public health crisis may affect mental health. Since individuals who are closer to a crisis are more likely to be affected by it, the anxiety and restlessness of these people should be higher. Thus, we propose the first hypothesis of this study:

**Hypothesis** **1:**  *The distance perception of a public health crisis is negatively associated with depression among people. The closer the perceived distance, the higher the depression of people.*

Risk perception can be divided into affective and cognitive risk perceptions. Because affective risk perception is strongly subjective and often irrational, it will increase the anxiety and restlessness of people. On the contrary, cognitive risk perception is based on the rational judgment of the public health crisis. This rational judgment is based on the scientific basis that is conducive to promoting the correct understanding of public health crises. Therefore, cognitive risk perception should reduce the anxiety and restlessness of people. Based on the above analysis, this study proposes the following two hypotheses:

**Hypothesis** **2:**  *Affective risk perception is positively associated with depression in public health crises. The higher the affective risk perception, the higher the depression.*

**Hypothesis** **3:**  *Cognitive risk perception is negatively associated with depression in public health crises. The higher the cognitive risk perception, the lower the depression.*

In a public health crisis, prevention and control policies play a significant role in relieving anxiety among people. Prevention and control policies at the central, local, and community levels can serve as social-emotional stabilizers. Generally, the effectiveness of prevention and control policies is reflected by the high recognition and support of these policies by the public. In addition, effective prevention and control policies are more likely to reduce public anxiety. On the contrary, ineffective prevention and control policies may lead the public to greater levels of anxiety and restlessness. Therefore, we propose the fourth hypothesis of this study:

**Hypothesis** **4:**  *Support for prevention and control policies is negatively associated with depression in public health crises. The stronger the support for prevention and control policies, the lower the depression.*

## 4. Research Design

### 4.1. Participants

The data used in this study were taken from a questionnaire survey project “Investigation on the Psychological Wellbeing and Behavioral Responses of the Public in the COVID-19 Crisis.” We aimed to investigate the psychological wellbeing and behavioral responses of adults in China during the COVID-19 crisis. Therefore, the targeted population of the study was people above 18 years old in China. The survey was organized from 29 February to 8 March 2020. Because the survey period occurred during the critical period of epidemic prevention and control, when the strictest quarantine policies were being implemented across China, household surveys with face-to-face interviews were impossible. In addition, we do not have a strict sampling frame. Therefore, the research team used a web platform to conduct an online survey. Although this strategy may cause the representation of the sampling to be biased, it helped the project team collect key information in the shortest amount of time, thus enabling countermeasures and suggestions for epidemic prevention and control to be proposed promptly.

Data were collected by cooperating with the wjx.cn company, one of the largest providers of online surveys in China. This company has over 83 million users and has collected more than 6.5 billion responses. Before the implementation of the survey, the project team optimized the structure of the questionnaire and item setting by consulting experts and conducting pilot surveys. In the formal phase of the online survey, if a respondent was younger than 18, the survey automatically ended. A total of 6373 people participated in the online survey, 1115 people completed the questionnaire, and the number of valid questionnaires was 1081. Table 1 shows that respondents were distributed across almost all of the provinces of China; Hubei, Hunan, and Guangdong were the three provinces with the largest number of respondents. This was not surprising because Hubei is at the center of the epidemic in China, Hunan is a neighboring province of Hubei, and Guangdong is the largest labor-exporting province for Hubei. The ethical principles of the Declaration of Helsinki were followed and ethical approval for this study was granted by the Ethics Committee of the corresponding author’s university (ethical approval number CCNU20LL007). Before the survey, we informed participants of the research purpose, assured them of the confidentiality of their personal information, and advised them of their rights to voluntarily participate in the survey.

### 4.2. Measurement

Depression (DEP) was measured using a 13-item scale (see Appendix A Table A1). The scale was developed based on the research of Derogatis and colleagues [47,48]. The responses of each item ranged from 1–5, representing never, seldom, sometimes, frequently, and all the time. A higher score indicates a higher level of DEP.

Distance perception (DIP) was measured using a 5-item scale (see Appendix A Table A2). The responses of each item ranged from 1–5, representing strongly disagree, disagree, so-so, agree, and strongly agree. A higher score suggests a closer DIP.

Affective risk perception (ARP) was measured using a 5-item scale (see Appendix A Table A3). The ARP scale was designed based on the research of Ferrer and colleagues [49]. The responses of each item ranged from 1–5, representing strongly disagree, disagree, so-so, agree, and strongly agree. A higher score indicates a stronger sense of ARP.

Cognitive risk perception (CRP) was measured with a 4-item scale (see Appendix A Table A4). The CRP scale was designed based on the research of Ferrer and colleagues [49]. The responses of each item ranged from 1–5, representing strongly disagree, disagree, so-so, agree, and strongly agree. A higher score implies a stronger sense of CRP.

Support for prevention and control policies (SPCP) was measured using a 6-item scale (see Appendix A Table A5). The responses of each item ranged from 1 to 5, representing strongly disagree, disagree, so-so, agree, and strongly agree. A higher score indicates a stronger sense of SPCP.

### 4.3. Methods

This study used structural equation modeling (SEM) to test the proposed hypotheses. In the first step, we reported the sampling characteristics of respondents. In the second step, we analyzed the reliability and validity of each measurement scale. Cronbach’s alpha coefficient, the Kaiser–Meyer–Olkin (KMO) test, and Bartlett’s test were used to examine the internal consistency of each scale. Exploratory factor analysis was performed to test the validity of each scale. In the third step, we analyzed the mental health status of respondents. In the fourth step, SEM was performed to identify the determinants of depression. We controlled for demographic and socioeconomic characteristics of respondents including gender, age, marital status, education, income, hukou (household registration), location (Wuhan or other places), and residential places (rural areas or urban areas) to strengthen the robustness of results. Data analysis was performed using Mplus 8.0 and Stata 14.0.

## 5. Results

### 5.1. Sampling Characteristics

Table 2 shows the demographic and socioeconomic characteristics of respondents. The percentages of males and females are 39% and 61%, respectively. The average age is about 32. With respect to the marital status of respondents, nearly half are married. With regard to the educational attainment of respondents, 8.42% have primary school and below education, 23.04% have middle school education, 32.47% have high school and polytechnic education, and 36% have university education or above. Regarding the income of respondents, about 28% of respondents’ income is less than 1000 yuan/month, 12% is between 1000 and 3000 yuan/month, 36% is between 3000 and 8000 yuan/month, and 24% is above 8000 yuan/month. Regarding hukou status, respondents with rural and urban hukou account for 39% and 61%, respectively. In addition, approximately 21% of respondents are residents of Wuhan city and 79.37% are non-Wuhan residents. Finally, about 68% of respondents reside in cities, and 32% reside in rural areas.

### 5.2. Test of the Reliability and Validity of the Measured Scales

We tested the internal reliability of the measurement scales by calculating the Cronbach’s alpha coefficients. To test the suitability of the scales for SEM, Kaiser‒Meyer‒Olkin (KMO) values were calculated and Bartlett’s test of measurement scales was performed. Table 3 shows the reliability and validity of the measured scales. The Cronbach’s alpha coefficient of the DEP scale is 0.959, the KMO value is 0.957, and the Bartlett test is highly significant (*p* < 0.001). Exploratory factor analysis shows that all items of the DEP scale belong to a common factor. The Cronbach’s alpha coefficient of the DIP scale is 0.948, the KMO value is 0.825, and the Bartlett test is highly significant (*p* < 0.001). Exploratory factor analysis shows that all items of the DIP scale belong to a common factor. The Cronbach’s alpha coefficient of the ARP scale is 0.842, the KMO value is 0.744, and the Bartlett test is highly significant (*p* < 0.001). Exploratory factor analysis shows that all items of the ARP scale belong to a common factor. The Cronbach’s alpha coefficient of the CRP scale is 0.891, the KMO value is 0.809, and the Bartlett test is highly significant (*p* < 0.001). Exploratory factor analysis shows that all items of the CRP scale belong to a common factor. The Cronbach’s alpha coefficient of the SPCP scale is 0.885, the KMO value is 0.887, and the Bartlett test is highly significant (*p* < 0.001). Exploratory factor analysis shows that all items of the SPCP scale belong to a common factor.

### 5.3. Depression of Respondents

**Figure 2:**
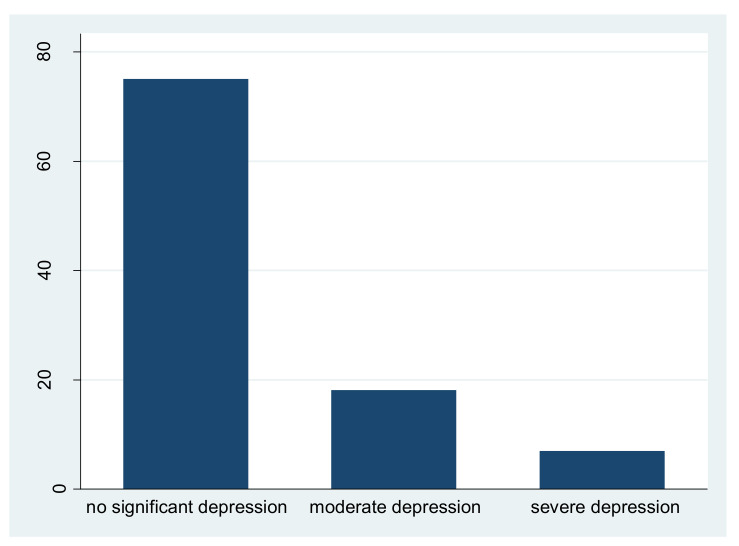
Mental health status of respondents.

Figure 2 shows the mental health status of respondents. We referred to previous studies [31,38,39] and determined that if the total score of the depression scale was between 13 and 26, no significant depression was identified. Moderate depression was identified if the total score of the depression scale was between 27 and 39. Severe depression was identified if the total score of the depression scale was above 40. Figure 2 shows that about 18% of respondents reported that they suffered moderate depression, and approximately 7% reported that they had very serious symptoms. This finding is similar to results of studies based on data collected in other countries. For instance, a survey in Italy found that about 22% of the sample had mental health problems [39], and a survey in Spain showed that about 25% of the sample had depression symptoms [38].

### 5.4. Structural Equation Modeling

**Figure 3:**
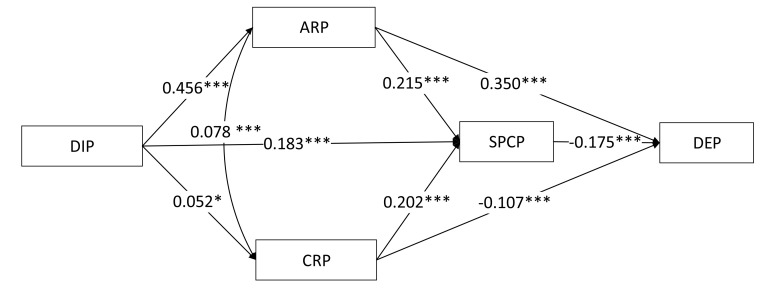
Results of structural equation modeling. Note: * *p* < 0.05, *** *p* < 0.001.

Table 4 shows a summary of the model fit of the SEM. Overall, the degree of model fit is very good. The χ2/df is 4.84, RMSEA is 0.060, SRMR is 0.045, CFI is 0.986, and TLI is 0.984. These indices suggest that the diamond model can fit the data very well. Figure 3 shows the standardized coefficients of the SEM. First, results show that DIP can indirectly affect mental health by influencing ARP, CRP, and SPCP. The total effect of DIP on depression is positive and significant. That is, for every one-unit increase in DIP, the depression of respondents increases by 0.103 unit. Thus, a closer DIP leads to a higher level of depression among respondents. Second, the direct effect of ARP on depression was positive and significant (β = 0.350, *p* < 0.001), the indirect effect is negative and significant (β = −0.038, *p* < 0.001), and the total effect is positive and significant (β = 0.313, *p* < 0.001). This means that for every one-unit increase in ARP, the depression of respondents increases by 0.313 unit. That is, the stronger the sense of ARP, the higher the level of depression of people. Third, the direct effect of CRP on depression is negative and significant (β = −0.107, *p* < 0.001), the indirect effect is also negative and significant (β = −0.035, *p* < 0.001), and the total effect is negative and significant (β = −0.142, *p* < 0.001). This means that CRP can remarkably reduce the depression of respondents. Finally, SPCP is negatively and significantly related to depression (β = −0.175, *p* < 0.001). For every one-unit increase in SPCP, the depression of respondents decreases by 0.175 units.

Notably, the results of SEM also show that DIP is negatively and significantly associated with ARP (β = 0.456, *p* < 0.001), CRP (β = 0.052, *p* < 0.001), and SPCP (β = 0.183, *p* < 0.001). This means that the closer the perception of the public health crisis, the stronger the sense of ARP, CRP, and SPCP of respondents. Moreover, both ARP and CRP are positively associated with SPCP. This finding suggests that when people have a high level of risk perception, they tend to support strict prevention and control policies, regardless of whether risk perception is affective or cognitive.

In a further analysis, we controlled for the demographic and socioeconomic characteristics of the respondents in SEM. Overall, the indices of model fit suggest that the revised model still fits the data very well. The χ2/df is 3.468, RMSEA is 0.048, SRMR is 0.051, CFI is 0.986, and TLI is 0.985. Results of the revised model are presented in Table 5. Regarding the association between demographic and socioeconomic characteristics and depression among respondents, age and location are significantly associated with depression. However, gender, marital status, education, income, hukou status, and residential places are not significantly associated with depression. Specifically, age is negatively related to depression (β = −0.152, *p* < 0.001). The higher the age, the lower the level of depression of respondents. Regarding the relationship between location and depression, respondents living in Wuhan have a significantly higher level of depression than respondents living in other places (β = 0.111, *p* < 0.001).

Generally, the effects of DIP, ARP, CRP, and SPCP on depression in the revised model are consistent with the results of the baseline model shown in Figure 3. First, the total effect of DIP on depression is still positive and significant (β = 0.109, *p* < 0.001), suggesting that the closer the distance perception of the public health crisis, the higher the depression of respondents. Therefore, the first hypothesis of this study cannot be rejected. Second, the total effect of ARP on depression is positive and significant (β = 0.314, *p* < 0.001). Thus, the second hypothesis cannot be rejected. Third, the total effect of CRP on depression is still negative and significant. Therefore, the third hypothesis of this study cannot be rejected. Finally, the effect of SPCP on depression is negative and significant (β = −0.135, *p* < 0.001). Hence, the fourth hypothesis of this study cannot be rejected.

## 6. Discussion

Although previous studies investigated how economic crises, natural disasters, and war and terrorist attacks affected mental health, the effect of public health crises on depression has yet to be explored. This study developed a “diamond model” that linked risk perception of a public health crisis to its associated factors, including distance perception of the crisis and support of prevention and control policies. Using online survey data on adults against the background of the COVID-19 crisis in China, this study empirically tested the hypotheses developed based on the diamond model.

First, this study finds that 25% of respondents reported moderate or severe depression symptoms. This figure is similar to the findings based on surveys in other countries such as Spain and Italy. This means that the psychological wellbeing of people globally is influenced by the COVID-19 crisis. Thus, the mental health problem of the public in the COVID-19 crisis is a global phenomenon and should be paid more attention.

Second, this study reveals that distance perception of the public health crisis is negatively associated with depression of people in public health crises. This finding is in line with the study of Lima, which reported a near-neighbor effect in which the mental health of people living closer to an incinerator is more likely to be affected by the incinerator [40]. In addition, Wu and colleagues found that, during the 2005 SARS event, respondents who were closer to SARS-related risks were more likely to report mental health problems [50]. Since epidemic diseases, such as COVID-19 and SARS, are highly contagious, people are very sensitive to highly infectious crowds and high-risk locations because living close to high-risk factors means that they are more likely to be infected. This may explain the negative association between DIP and depression. Results of SEM also suggest that DIP is significantly associated with ARP, CRP, and SPCP. Respondents who have a closer DIP tended to show higher risk perception, both affectively and cognitively. In addition, respondents who have a closer DIP tend to be more likely to support strict prevention and control policies. These two findings reflect that people are more sensitive to the public health crisis when they feel that they live close to high-risk places.

Third, this study shows that while ARP is positively related to depression among respondents, CRP is negatively related to depression among respondents. This finding confirms a conclusion of previous studies, namely, that it is highly important to categorize different types of risk perception [43,44,45]. Furthermore, this finding means that ARP and CRP affect respondents’ mental health in totally different ways. Because ARP is a type of heuristic information processing that is fast, intuitive, parallel, and spontaneous, it is a very subjective and irrational type of judgment. Thus, the psychological wellbeing of people in a public health crisis is negatively affected by ARP. In contrast, CRP is a type of information processing that is slow, cautious, sequential, and controllable. Rational cognition is able to help people calm their emotions in public health crises. In addition, this finding means that it is very important to promote the CRP and reduce the ARP of citizens through various interventions in a public health crisis.

Fourth, this study suggests that SPCP is negatively associated with depression among respondents. A recent study showed that quarantine policies have a negative impact on the mental health of university students [1]. However, the present study shows that if prevention and control policies are effective and supported by people, they can effectively reduce the psychological distress experienced during a public health crisis. A possible explanation for these differences is that quarantine measures usually cause not only physical distancing but also social distancing. While physical distancing may help reduce the spread of the COVID-19 virus, social distancing is detrimental to psychological wellbeing. If prevention and control policies can enforce physical distancing without causing serious social distancing, people will generally regard these policies as a type of social support rather than a social control. Previous studies have shown the importance of social and policy support in alleviating the depression of people who suffer crisis events [2,6]. The negative association between SPCP and depression may hint that if people in public health crises support prevention and control policies, they will regard these policies as a sort of social support.

The theoretical implication of this study is that when analyzing how a public health crisis affects the mental health of people, it is important to consider the role of risk perceptions. In particular, risk perceptions should be divided into ARP and CRP because these two types of risk perceptions have distinctive effects on the mental health of people during public health crises. In addition, associated factors of risk perception, including DIP and SPCP, also play a significant role in determining the mental health of people during a public health crisis. DIP significantly affects ARP, CRP, and SPCP, and ARP and CRP significantly influence SPCP. Moreover, DIP, ARP, CRP, and SPCP all significantly affect the mental health of people in a public health crisis. This justifies the diamond model of this study, which links the risk perception of the public health crisis to DIP and SPCP. Therefore, health policies aiming to improve the psychological wellbeing of people in a public health crisis should take risk perception into consideration. Moreover, this model may be extended to predict the mental health of people in other types of public health crises.

This study also has policy implications. First, the negative association between DIP and depression means that, in a public health crisis, people generally require a safe sense of distance. Thus, it is important to enforce physical (but not social) distancing measures in the COVID-19 crisis. Physical isolation measures are useful for blocking the spread of the virus and reducing the possibility of citizens being affected [51]. Measures such as these may increase the sense of security of the public. As a result, they are less likely to be depressed by the COVID-19 crisis. Second, the distinctive effects of ARP and CRP on depression mean that when a public health crisis happens, it is vital to promote health education among the public. Health education can increase the scientific recognition of the public health crisis among the public. This strategy may effectively reduce ARP and increase CRP of the public, which could help to reduce their anxiety and restlessness. Third, the negative association between SPCP and depression means that it is necessary to conduct policy promotion when implementing the prevention and control policies. When an epidemic prevention policy is well understood by the public, they will tend to support the policies, which is beneficial to their psychological wellbeing.

This study has some limitations. First, data were taken from an online survey that was not based on representative sampling. This issue may cause the empirical analysis to be biased. In addition, this study only collected one wave of survey data. In a public health crisis, people may gradually adapt to the crisis over time; that is, people’s depression may gradually decrease with time. Therefore, a longitudinal research design may better reveal the psychological condition of people during a public health crisis. Moreover, other important issues, such as social capital, community support, and medical resources, are significant factors that may influence the mental health of people during a public health crisis. However, these factors were not included in the empirical analysis of our study. Our future study will be dedicated to revealing the mental health of people in a public health crisis using a more elaborate research design.

## 7. Conclusions

The results show that risk perception and its associated factors significantly affect the mental health of people in public health crises. Specifically, DIP of public health crises is negatively associated with depression among people. In addition, ARP is positively associated with depression of people in public health crises. Moreover, CRP is negatively associated with depression of people in public health crises. Furthermore, SPCP is negatively associated with depression of people in public health crises. These results suggest that risk perception plays an important role in affecting the mental health of people in a public health crisis. Therefore, health policies aiming to improve the psychological wellbeing of the people in a public health crisis should take risk perception into consideration.

## Figures and Tables

**Table 1:** Distribution of the sample.

Provinces	Respondents	Percentage
Hubei	464	42.92
Hunan	126	11.66
Guangdong	70	6.48
Anhui	48	4.44
Henan	46	4.26
Beijing	36	3.33
Sichuan	36	3.33
Jiangsu	34	3.15
Shandong	32	2.96
Zhejiang	22	2.04
Shanxi	18	1.67
Ningxia	18	1.67
Chongqing	16	1.48
Guangxi	16	1.48
Jiangxi	14	1.30
Hainan	10	0.93
Heibei	9	0.83
Shanghai	9	0.83
Xinjiang	9	0.83
Shaanxi	8	0.74
Fujian	8	0.74
Yunan	8	0.74
Tianjin	5	0.46
Inner Mongolia	4	0.37
Guizhou	4	0.37
Heilongjiang	4	0.37
Gansu	3	0.28
Liaoning	2	0.19
Jilin	2	0.19

**Table 2:** Descriptive statistics of sampling characteristics.

Variables	Obervations	Percentage/Mean
Gender		
Male	420	38.85%
Female	661	61.15%
Age (standard divation)	1081	32.58 (11.00)
Marital status		
Unmarried	531	49.12%
Married	550	50.88%
Education		
Primary school and below	91	8.42%
Middle school	249	23.04%
High schol or polytech	351	32.47%
University or above	390	36.07%
Income		
Less than 1000 yuan/month	302	27.94%
1000–3000 yuan/month	128	11.84%
3000–8000 yuan/month	394	36.45%
More than 8000 yuan/month	257	23.77%
Hukou		
Rural hukou	421	38.95%
Urban hukou	660	61.05%
Location		
Wuhan	223	20.63%
Non-Wuhan	858	79.37%
Residence		
Rural areas	350	38.95%
Urban areas	731	67.62%

**Table 3:** Reliability and validity of measure scales.

Scales	Item	Factor Loadings	KMO	Bartlett Test	Cronbach’s Alpha	Mean	SD
DEP	DEP1	0.816	0.959	*p* < 0.001	0.957	1.729	0.798
	DEP2	0.801					
	DEP3	0.817					
	DEP4	0.766					
	DEP5	0.684					
	DEP6	0.846					
	DEP7	0.822					
	DEP8	0.888					
	DEP9	0.879					
	DEP10	0.791					
	DEP11	0.846					
	DEP12	0.823					
	DEP13	0.828					
DIP	DIP1	0.890	0.825	*p* < 0.001	0.948	3.792	0.920
	DIP2	0.932					
	DIP3	0.957					
	DIP4	0.925					
	DIP5	0.843					
ARP	ARP1	0.702	0.744	*p* < 0.001	0.842	3.751	0.792
	ARP2	0.795					
	ARP3	0.772					
	ARP4	0.823					
	ARP5	0.825					
CRP	CRP1	0.878	0.809	*p* < 0.001	0.891	3.512	0.912
	CRP2	0.920					
	CRP3	0.872					
	CRP4	0.806					
SPCP	SPCP1	0.892	0.887	*p* < 0.001	0.885	4.337	0.631
	SPCP2	0.860					
	SPCP3	0.871					
	SPCP4	0.893					
	SPCP5	0.577					
	SPCP6	0.803					

Note: DEP—Depression; DIP—Distance perception; ARP—Affective risk perception; CRP—Cognitive risk perception; SPCP—Support for prevention and control policies; KMO—Kaiser‒Meyer‒Olkin; SD—Standard Deviation.

**Table 4:** Summary of the model fit of structural equation modeling.

Indices of Model Fit	Standard	Model Fit	Results
χ2/df	<5.00	4.840	good
RMSEA	<0.08	0.060	good
SRMR	<0.08	0.045	good
CFI	>0.90	0.986	good
TLI	>0.90	0.984	good

Note: χ2/df refers to the ratio of Chi-square value to degrees of freedom. RMSEA refers to root mean square error of approximation. SRMR refers to standardized root mean square residual. CFI refers to comparative fit index. TLI refers to Tucker-Lewis index.

**Table 5:** Standardized coefficients of the revised structural equation modeling.

Path	Coefficients	SE	*p*-Value
Gender → DEP	−0.060	0.033	0.066
Age → DEP	−0.152	0.042	0.000
Marriage → DEP	−0.052	0.042	0.216
Education → DEP	0.044	0.035	0.202
Income → DEP	−0.038	0.038	0.309
Hukou → DEP	−0.079	0.042	0.057
Wuhan → DEP	0.111	0.033	0.001
Rural → DEP	0.063	0.040	0.122
ARP → DEP	0.311	0.032	0.000
CRP → DEP	−0.099	0.030	0.001
SPCP → DEP	−0.192	0.035	0.000
DIP → SPCP	0.157	0.036	0.000
ARP → SPCP	0.197	0.038	0.000
CRP → SPCP	0.197	0.031	0.000
DIP → ARP	0.568	0.022	0.000
DIP → CRP	0.061	0.028	0.032

## Appendix A

**Table A1:** Scale to measure depression of the public.

Items	1	2	3	4	5
I experienced outbursts of anger that I could not control (DEP1)	Never	Seldom	Sometimes	Frequently	All the time
I yelled at somebody or threw things (DEP2)	Never	Seldom	Sometimes	Frequently	All the time
I had a row with someone (DEP3)	Never	Seldom	Sometimes	Frequently	All the time
I was easily annoyed and irritated (DEP4)	Never	Seldom	Sometimes	Frequently	All the time
I wanted to break or damage things (DEP5)	Never	Seldom	Sometimes	Frequently	All the time
I felt lonely (DEP6)	Never	Seldom	Sometimes	Frequently	All the time
The future seemed hopeless (DEP7)	Never	Seldom	Sometimes	Frequently	All the time
I had sleeping problems (DEP8)	Never	Seldom	Sometimes	Frequently	All the time
I was sad or had little interest in doing things (DEP9)	Never	Seldom	Sometimes	Frequently	All the time
I felt sad or blue (DEP10)	Never	Seldom	Sometimes	Frequently	All the time
I was not excited in doing things (DEP11)	Never	Seldom	Sometimes	Frequently	All the time
I cried easily or wanted to cry (DEP12)	Never	Seldom	Sometimes	Frequently	All the time
I was slow or had little energy (DEP13)	Never	Seldom	Sometimes	Frequently	All the time

**Table A2:** Scale to measure distance perception of the COVID-19 crisis.

Items	1	2	3	4	5
I am afraid that there are confirmed patients of COVID-19 in my city (DIP1)	Strongly disagree	Disagree	So-so	Agree	Strongly agree
I am afraid that there are confirmed patients of COVID-19 in my county (district) (DIP2)	Strongly disagree	Disagree	So-so	Agree	Strongly agree
I am afraid that there are confirmed patients of COVID-19 in my town (street) (DIP3)	Strongly disagree	Disagree	So-so	Agree	Strongly agree
I am afraid that there are diagnosed patients of COVID-19 in my community (villages) (DIP4)	Strongly disagree	Disagree	So-so	Agree	Strongly agree
I am afraid that my neighbor has a confirmed patient of COVID-19 (DIP5)	Strongly disagree	Disagree	So-so	Agree	Strongly agree

**Table A3:** Scale to measure affective risk perception.

Items	1	2	3	4	5
I am worried about the possible consequences of COVID-19 (ARP1)	Strongly disagree	Disagree	So-so	Agree	Strongly agree
I am afraid of the possible consequences of COVID-19 (ARP2)	Strongly disagree	Disagree	So-so	Agree	Strongly agree
I hate the possible consequences of COVID-19 (ARP3)	Strongly disagree	Disagree	So-so	Agree	Strongly agree
I am dissatisfied with the possible consequences of COVID-19 (ARP4)	Strongly disagree	Disagree	So-so	Agree	Strongly agree
I am angry about the possible consequences of COVID-19 (ARP5)	Strongly disagree	Disagree	So-so	Agree	Strongly agree

**Table A4:** Scale to measure cognitive risk perception.

Items	1	2	3	4	5
Because I (and my family members) pay great attention to the epidemic, I think we have a low chance of being infected by COVID-19 (CRP1)	Strongly disagree	Disagree	So-so	Agree	Strongly agree
Because I (and my family members) have a good lifestyle, I think we have a low chance of being infected by COVID-19 (CRP2)	Strongly disagree	Disagree	So-so	Agree	Strongly agree
Because I (and my family members) know professional protection knowledge, I think we have a low chance of being infected by COVID-19 (CRP3)	Strongly disagree	Disagree	So-so	Agree	Strongly agree
Because I (and my family members) am in good health, I think we have a low chance of being infected by COVID-19 (CRP4)	Strongly disagree	Disagree	So-so	Agree	Strongly agree

**Table A5:** Scale to measure support for prevention and control policies.

Items	1	2	3	4	5
It is necessary for the government to take the measure of seal off the city (SPCP1)It is necessary for the government to take the measure of road closure (SPCP2)	Strongly disagree	Disagree	So-so	Agree	Strongly agree
It is necessary for the government to close all businesses and entertainment venues (SPCP3)	Strongly disagree	Disagree	So-so	Agree	Strongly agree
It is necessary for the government to take the strategy of closed management of the community (SPCP4)	Strongly disagree	Disagree	So-so	Agree	Strongly agree
It is necessary for the government to send staff to each household for temperature testing (SPCP5)	Strongly disagree	Disagree	So-so	Agree	Strongly agree
It is necessary for the government to monitor the temperature of passengers at stations and ports (SPCP6)	Strongly disagree	Disagree	So-so	Agree	Strongly agree
